# The Distant Molecular Effects on the Brain by Cancer Treatment

**DOI:** 10.3390/brainsci14010022

**Published:** 2023-12-24

**Authors:** Kimberly Demos-Davies, Jessica Lawrence, Clara Ferreira, Davis Seelig

**Affiliations:** 1 Department of Veterinary Clinical Sciences, University of Minnesota College of Veterinary Medicine, Saint Paul, MN 55108, USA; jlawrenc@umn.edu (J.L.); dseelig@umn.edu (D.S.); 2 Masonic Cancer Center, University of Minnesota, Minneapolis, MN 55455,USA; 3 Department of Radiation Oncology, University of Minnesota Medical School, Minneapolis, MN 55455, USA; cferreir@umn.edu

**Keywords:** cancer treatment, cancer-related cognitive impairment CRCI, cancer neuropathology, SKH1 mice

## Abstract

Cancer survivors experience cancer-related cognitive impairment (CRCI) secondary to treatment. Chemotherapy and radiation therapy independently contribute to cognitive dysfunction; however, the underlying mechanisms leading to dysfunction remain unclear. We characterized brain gene expression changes in a mouse model of CRCI to identify the mechanistic underpinnings. Eleven-to-twelve-week-old SKH1 mice were treated with doxorubicin (DOX), hindlimb radiation (RT), concurrent hindlimb radiation and doxorubicin (DOX-RT), or no treatment (control). Sixteen days following treatment, gene expression was measured from murine brains using the NanoString nCounter^®^ glial profiling panel. Gene expression was normalized and compared between groups. No two groups shared the same expression pattern, and only *Gnb1* and *Srpr* were upregulated in multiple treatment groups. Brains from DOX-treated mice had upregulated *Atf2*, *Atp5b*, *Gnb1*, *Rad23b*, and *Srpr* and downregulated *Sirt5* expression compared to control brains. Brains from RT-treated mice demonstrated increased *Abcg2* and *Fgf2* and decreased *C1qa* and *C1qb* expression compared to control brains. Brains from DOX-RT-treated mice had upregulated *Adar*, *E2f3*, *Erlec1*, *Gnb1*, *Srpr, Vim*, and *Pdgfra* expression and downregulated *Rock2* and *Inpp5f* expression compared to control brains. The gene expression changes demonstrated here highlight roles for neuronal transmission and oxidative stress in the pathogenesis of doxorubicin-related CRCI and inflammation in RT-related CRCI.

## 1. Introduction

Improvements in cancer detection and treatment have increased the number of cancer survivors with long-term survival [1,2,3,4]. Although the lifespan of the average cancer patient has increased, the adverse effects of cancer treatment can be detrimental to long-term quality of life. Cancer-related cognitive impairment (CRCI) affects patients with and without central nervous system cancer and significantly contributes to survivorship challenges. CRCI is a neurocognitive syndrome estimated to affect up to 75% of cancer patients, continuing for 5–10 years following treatment [1,2,5]. CRCI is clinically defined by learning and memory deficits and impaired concentration, processing speed, and executive function [1,5,6]. Cancer survivors’ quality of life is detrimentally impacted by CRCI, and it negatively affects their ability to return to work, perform daily tasks, and maintain their personal relationships [7].

CRCI is particularly well documented in breast cancer survivors, as treatment improvements have resulted in a 90% five-year survival rate [8]. Breast cancer patients are frequently prescribed chemotherapy and radiation therapy, both of which have been linked to CRCI [1,2,9,10]. Imaging studies in female breast cancer patients treated with chemotherapy have revealed decreases in grey matter volume, reductions in white matter microstructure, neuroinflammation, altered cerebral blood flow, and changes in brain connectivity [4,9,10,11,12,13]. Several proposed mechanisms leading to neurocognitive symptoms after chemotherapy include disruption of the blood–brain barrier, neuronal apoptosis, decreased neurogenesis, oxidative stress, myelin degeneration, DNA damage, genetic predispositions, altered brain blood flow, and cytokine dysregulation [1,2,3].

Radiation therapy is a standard-of-care treatment for multiple cancers, and over 50% of all newly diagnosed cancer patients will receive radiation therapy during their course of treatment [2]. The direct effects of radiation therapy on the brain include cognitive impairment, white matter necrosis, vascular changes, demyelination, and neuroinflammation [14,15]. Much less is known regarding the ability of radiation to induce cognitive deficits when anatomic sites distant to the brain are irradiated. Previous reports have documented persistent impairment in memory and executive function following non-brain-directed radiation therapy [2,13].

The 2021 GLOBOCAN cancer burden survey estimated that 19.3 million new cancer cases occurred in 2020 and that this number will grow to 28.4 million in 2040 [16]. This increase in cancer incidence will result in increased usage of therapeutic chemotherapy and radiation. It is estimated by the year 2040, there will be 15 million cancer patients requiring chemotherapy, and by the year 2030, there will be 4.17 million 5-year cancer survivors who received radiation therapy [17,18]. Given this increase in chemotherapy- and radiation-treated cancer patients and improved cancer outcomes, the characterization of the role of cancer treatment in cancer patient survivorship issues, including CRCI, is an urgent need.

The exact mechanisms causing chemotherapy- and/or radiotherapy-induced CRCI are not understood, which hinders solutions to mitigate its occurrence. In prior work, we demonstrated that the studied mice developed multifocal brain gliosis and hippocampal memory deficits when treated with doxorubicin, hindlimb radiation, or the concurrent administration of doxorubicin and radiation [1]. To shed mechanistic insight on the link between non-brain-directed radiation, brain gliosis, and cognitive impairment, we performed gene expression profiling in the brains of mice treated with doxorubicin, non-brain-directed radiation, or concurrent doxorubicin and radiation using the NanoString nCounter^®^ platform (NanoString Technologies, Seattle, WA, USA).

## 2. Materials and Methods

### 2.1. Experimental Animals

Eleven-to-twelve-week-old female SKH1 mice were purchased from Charles River Laboratories (Wilmington, MA, USA). As female breast cancer survivors are disproportionately affected by CRCI, only female mice were used in this study [19]. To isolate treatment-associated changes from tumor changes, non-tumor-bearing mice were used in this study [1,5,9,13]. Mice were assigned to one of four groups according to body weight: (1) mice treated with doxorubicin only (DOX), (2) mice treated with non-brain-directed radiation treatment (RT), (3) mice treated with both (DOX-RT), and (4) untreated mice (control). Mice were housed in static cages, with four female mice from the same treatment group per cage. This study was performed with approval by and in accordance with the University of Minnesota Institutional Animal Care and Use Committee (UMN-IACUC).

### 2.2. Animal Treatment

The therapeutic methodology was identical to our previous study [1]. Briefly, doxorubicin HCL (Hikma Pharmaceuticals USA Inc., Berkeley Heights, NJ, USA) was administered intraperitoneally at 5 mg/kg. We chose to administer a single dose of 5 mg/kg (equivalent to 18 mg/m^2^), which is lower than the claimed lethal dose in mice of 7–10 mg/kg IP and comparable to doxorubicin dosages (10–20 mg/m^2^) administrated with radiation therapy in human cancer patients [20,21,22,23]. The radiation protocol performed on the RT and DOX-RT mice consisted of 20 Gy applied to the skin of the right hindlimb with 6 MeV electrons (Varian 2100 iX; Varian Medical Systems, Inc., Palo Alto, CA, USA) using a 1 cm tissue-equivalent bolus and a 2 × 2 cm^2^ electron cutout [1]. The dose of radiation was quantified using radiochromic film dosimetry (GAFchromicTM EBT2, Ashland AdvancedMaterials, Bridgewater, NJ, USA) to confirm the prescribed dose was given to the right hindlimb and that the dose was undetectable at the level of the skin over the skull [24]. The dose of radiation administered was comparable to the dose recommended to be given over 7 days in early-stage breast cancer patients [25]. Control mice were anesthetized with xylazine (4 mg/kg) and ketamine (90 mg/kg) and treated with intraperitoneal saline. Cognitive deficits were analyzed through standardized behavioral testing, including the open field test, novel location recognition test, novel object recognition test, and spontaneous alternation y-maze, prior to collection of brain tissue [1].

### 2.3. Tissue Preparation

Sixteen days post treatment, mice were euthanized by carbon dioxide followed by exsanguination in accordance the UMN-IACUC Criteria for Carbon Dioxide Euthanasia Guidelines. This timepoint was selected to evaluate acute-term effects of cancer therapy translational to almost 2 years post cancer treatment in adult humans [26]. Mouse brains were collected, immersion-fixed in 10% neutral buffered formalin, and transferred into 70% ethanol. Samples were sectioned and subjected to routine tissue processing before paraffin embedding.

### 2.4. NanoString Gene Expression Profiling

Brain tissues from six mice from each treatment group were processed for gene expression profiling. The brains of mice were split into two paraffin blocks. A total of twenty 10 µm coronal sections of the brains were collected for RNA extraction. The first coronal sections from two paraffin blocks of each brain included the caudal cortex (coronal sections near −3.38 to −4.08 Bregma), cerebellum (coronal sections near −6.355 to −7.255 Bregma), hippocampus (coronal sections near −2.78 to −3.455 Bregma), medulla (coronal sections near −6.355 to −7.255 Bregma), midbrain (coronal sections near −3.38 to −4.08 Bregma), rostral cortex (coronal sections near −0.08 to −1.455 Bregma), and striatum (coronal sections near −0.08 to −1.455 Bregma) [1,27]. RNA was extracted using the PureLink FFPE total RNA isolation kit (Invitrogen, Carlsbad, CA, USA) according to manufacturer’s instructions. A total of 100 nanograms of RNA from each sample was used to evaluate gene expression by the nCounter^®^ glial cell profiling panel from NanoString (NanoString Technologies, Seattle, WA, USA).

### 2.5. Data Analysis

The nSolver™ Analysis Software version 4.0 (NanoString Technologies, Seattle, WA, USA) quality control parameters were used to assess quality of imaging, binding density, positive control linearity, and positive control limit of detection. All samples passed the quality controls assessed [28,29]. The nSolver™ Analysis Software Advanced Analysis Module (version 2.0.134) was used to analyze gene expression data including gene normalization as well as differential expression volcano plots with *p*-values from linear regression [30]. The Advanced Analysis Module was used to normalize raw gene data for each sample to the geometric mean of the endogenous housekeeping genes using the geNorm algorithm [28,29]. The normalized gene expression data were analyzed using Prism 10.1.2 (GraphPad Software, San Diego, CA, USA). One-way analysis of variance (ANOVA) with the Tukey post hoc test was used to evaluate differences between groups.

A pathway enrichment analysis was performed using Enrichr on the 17 genes differentially expressed in the brains of mice treated with cancer therapy compared to control mice [31,32,33]. The databases included were *BioPlanet 2019*, *WikiPathway 2023 Human*, *KEGG 2021 Human*, and *Elsevier Pathway Collection*. From each database, the top 10 significant *p*-values enrichment results were reported.

## 3. Results

### Cancer Treatment Is Associated with Unintended Molecular Changes in the Normal Brain

**Figure 1:**
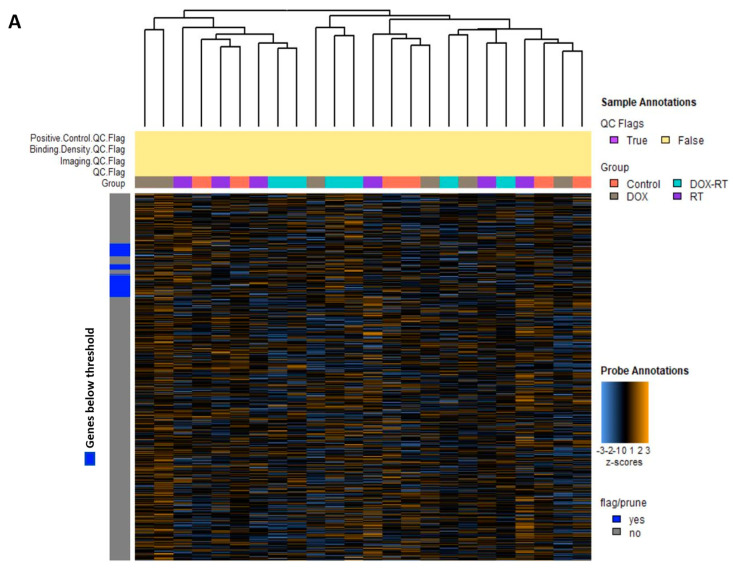

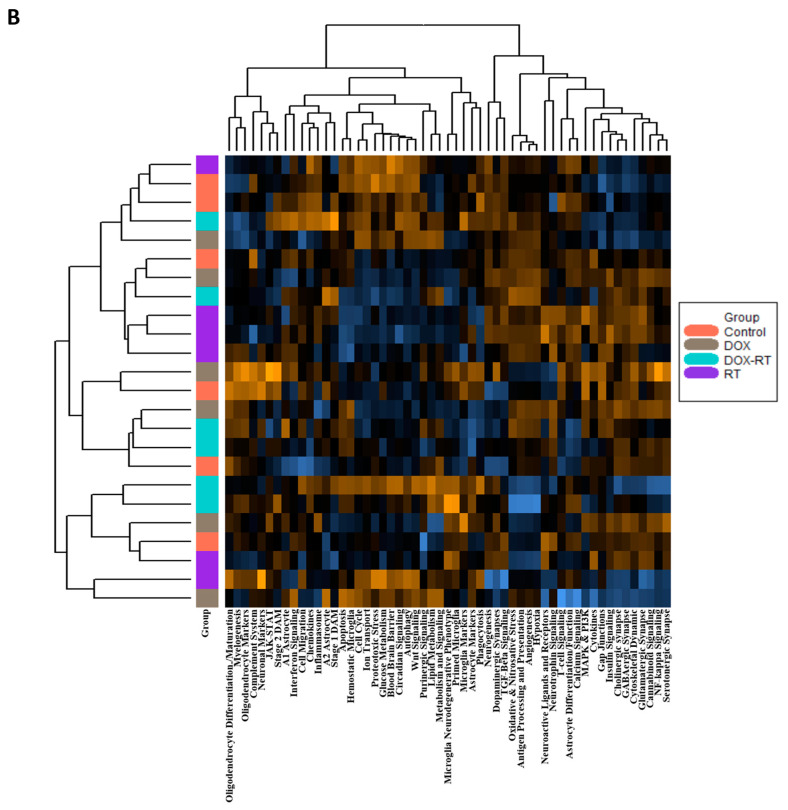
Overview of gene changes in the brain of mice treated with cancer treatment. (**A**) Heat map of normalized data, with orange indicating high expression and blue indicating low expression. (**B**) Heatmap showing gene pathway clustering of the groups. Control, control mice; DOX, doxorubicin-treated mice; RT, hindlimb-radiation-treated mice; DOX-RT, doxorubicin- and hindlimb-radiation-treated mice. *n* = 6 mice per group.

Glial cell activation is associated with worse cognitive impairment in breast cancer patients after cancer treatment [4]. We previously found widespread glial cell activation and cognitive impairment following cytotoxic therapy in this mouse model [1]. To better understand molecular signals associated with chemotherapy, radiation, or concurrent treatment, we used the nCounter^®^ glial cell profiling panel to characterize gene expression patterns in the brains of normal control mice and DOX-, RT-, and DOX-RT-treated mice. Unsupervised hierarchical clustering of the normalized gene expression data for all mice (Figure 1A) and the heat map of gene pathway cluster scores (Figure 1B) demonstrated unique changes to all treated groups.

**Figure 2:**
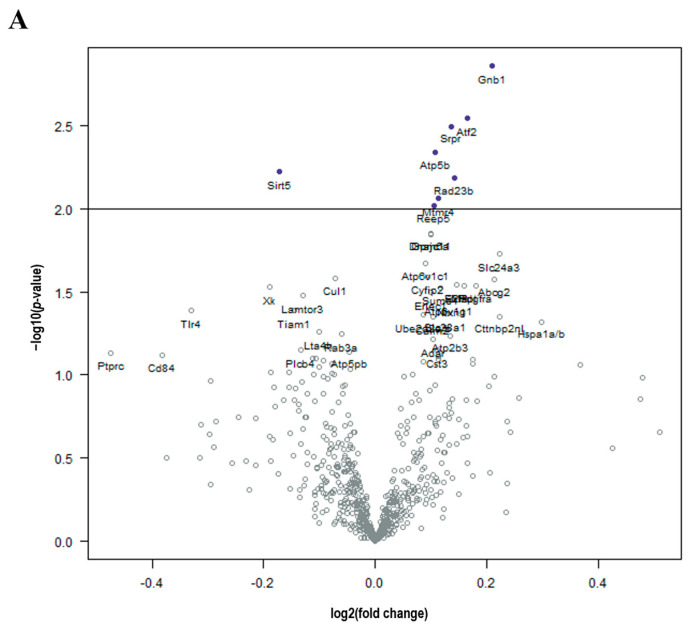

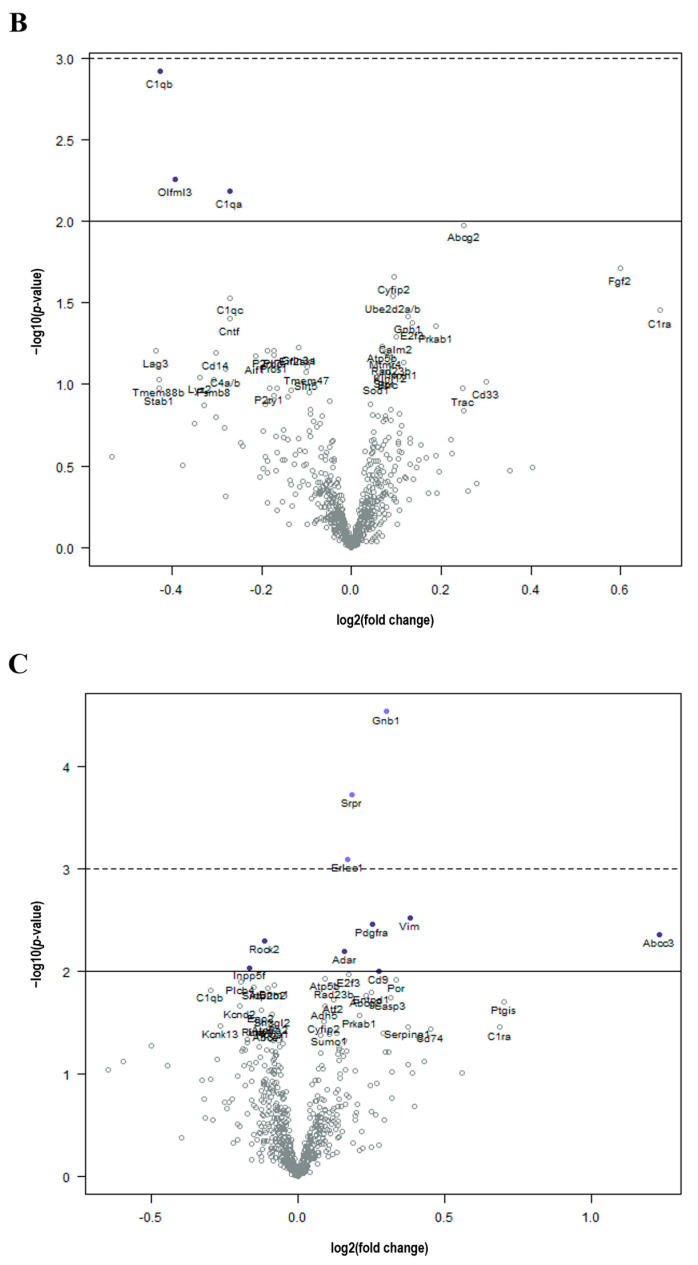
Cancer treatment is associated with molecular changes in the brain. (**A**) Volcano plot shows all differentially expressed genes above background between brains from DOX and control mice, with genes of high statistical significance on top and high fold change on either side using a log linear regression. (**B**) Volcano plot shows all differential expressed genes above background between brains from RT and control mice, with genes of high statistical significance on top and high fold change on either side using a log linear regression. (**C**) Volcano plot shows all differential expressed genes above background between brains from DOX-RT and control mice, with genes of high statistical significance on top and high fold change on either side using a log linear regression. Solid line *p*-value < 0.01; dotted line *p*-value < 0.001. Control, control mice; DOX, doxorubicin-treated mice; RT, hindlimb-radiation-treated mice; DOX-RT, doxorubicin- and hindlimb-radiation-treated mice. n = 6 mice per group.

**Figure 3:**
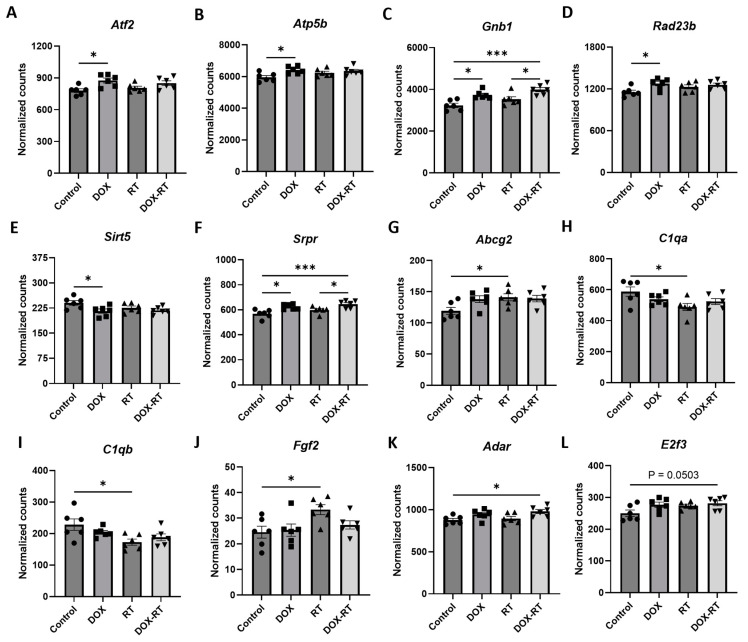

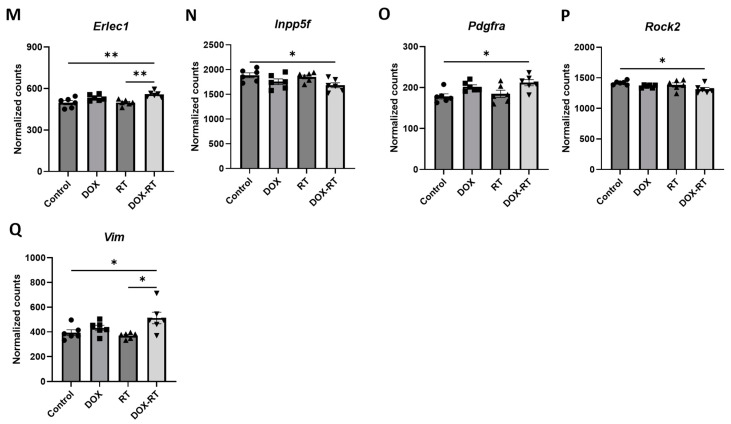
Cancer treatment causes significant gene changes in the brain of mice. Mice treated with DOX had significant upregulation of genes in their brain, including *Atf2* (**A**), *Atp5b* (**B**), *Gnb1* (**C**), *Rad23b* (**D**), and *Srpr* (**F**), compared to control mice. Mice treated with DOX-RT had significant downregulation of the *Sirt5* (**E**) gene in their brain compared to control mice. Comparison showed brains from mice treated with RT had significant upregulation of *Abcg2* (**G**) and *Fgf2* (**J**) genes and downregulation of *C1qa* (**H**) and *C1qb* (**I**) compared to control mice. Mice treated with DOX-RT had significant upregulation of *Adar* (**K**), *E2f3* (**L**), *Erlec1* (**M**), *Gnb1* (**C**), *Pdgfra* (**O**), *Srpr* (**F**), and *Vim* (**Q**) gene expression and downregulation of *Inpp5f* (**N**) and *Rock2* (**P**) gene expression compared to control mice. Data represent the mean and SEM evaluated by ANOVA and post hoc analysis. DOX, doxorubicin-treated group; RT, hindlimb-radiation-treated group; DOX-RT, doxorubicin- and hindlimb-radiation-treated group. n = 6 mice per group. * *p*-value < 0.05; ** *p*-value < 0.01; *** *p*-value < 0.001.

Brain tissue from DOX-treated mice demonstrated significant (*p*-value < 0.01) upregulation in *Gnb1*, *Atf2*, *Srpr*, *Atp5b*, *Rad23b*, and *Mtmr4* and downregulation in *Sirt5* compared to control in a log linear regression model (Figure 2A). Upon ANOVA and post hoc analysis, DOX-treated brains had significantly upregulated *Atf2* (*p* = 0.0130, Figure 3A), *Atp5b* (*p* = 0.0245, Figure 3B), *Gnb1* (*p* = 0.0105, Figure 3C), *Rad23b* (*p* = 0.0298, Figure 3D) and *Srpr* (*p* = 0.0137, Figure 3F) gene expression compared to control brains. Only *Sirt5* (*p* = 0.0341, Figure 3E) expression was downregulated compared to the control.

Compared to control mice, brains from mice treated with RT demonstrated significant (*p*-value < 0.01) decreased expression of *C1qb*, *Olfml3*, and *C1qa* in a log linear regression model (Figure 2B). Upon ANOVA and post hoc analysis, the upregulated genes from RT-treated brains as compared to the control included *Abcg2* (*p* = 0.0495, Figure 3G) and *Fgf2* (*p* = 0.0350, Figure 3J). The downregulated genes as compared to control were *C1qa* (*p* = 0.0270, Figure 3H) and *C1qb* (*p* = 0.0190, Figure 3I).

As compared to control mice, mice treated with DOX-RT demonstrated significant (*p*-value < 0.01) increased expression of *Gnb1*, *Srpr*, *Erlec1*, *Vim*, *Pdgfra*, *Abcc3*, *Adar*, and *CD9* and decreased expression of *Rock2* and *Inpp5f* in a log linear regression model (Figure 2C). Upon ANOVA and post hoc analysis, upregulated *Adar* (*p* = 0.0278, Figure 3K), *E2f3* (*p* = 0.0503, Figure 3L), *Erlec1* (*p* = 0.0034, Figure 3M), *Gnb1* (*p* = 0.0002, Figure 3C), *Pdgfra* (*p* = 0.0147, Figure 3O), *Srpr* (*p* = 0.0008, Figure 3F), and *Vim* (*p* = 0.0377, Figure 3Q) expression was measured compared to control brains. Only two genes were downregulated (Figure 3), including *Inpp5f* (*p* = 0.0442, Figure 3N) and *Rock2* (*p* = 0.0252, Figure 3P).

When comparing treatment groups, brains from DOX-treated mice did not differ in gene expression from RT- or DOX-RT-treated mice. RT-treated mice had significant gene expression changes in their brains compared to DOX-RT-treated mice (Table 1). RT-treated mice had increased expression of *Atp2b2* compared to DOX-RT treated mice and decreased *Cd74*, *Erlec1* (Figure 3M), *Gnb1* (Figure 3C), *Lyz2*, *Olfml3*, *Srpr* (Figure 3F), and *Vim* (Figure 3Q) expression compared to DOX-RT-treated mice (Table 1).

A pathway enrichment analysis was performed on the 17 differentially expressed genes in the DOX-, RT-, and DOX-RT-treated mice compared to the control (Table 2). The 40 pathway enrichments included 13 associated with cancer and 5 associated with infectious agents. Other functions included in the enrichment analysis were complement cascade activation, cytoskeleton regulation, angiogenesis, and oxidative stress (Table 2).

## 4. Discussion

In this study, we sought to identify gene expression changes in the brains of a previously published mouse model of CRCI to better understand the mechanistic links between cognitive dysfunction and distinctive cancer therapies, namely focal radiation treatment and systemic doxorubicin. The main overall findings of this study highlight roles for neuronal transmission and oxidative stress in the pathogenesis of doxorubicin-related CRCI and inflammation in RT-related CRCI. This study builds upon our previous work demonstrating that these mice show extensive brain gliosis and hippocampal-dependent memory deficits after treatment with either hindlimb radiation, doxorubicin, or concurrent treatment [1]. The results highlight the importance of documenting distinct, treatment-specific mechanisms to define potential mitigators for CRCI, as our data demonstrated differentially expressed gene changes in the brains of SKH1 mice after treatment with DOX, RT, or DOX-RT compared to control mice. In addition, we observed significant gene expression changes between the RT-treated mice and DOX-RT-treated mice. Surprisingly, neither RT- nor DOX-treated mice shared similar gene expression patterns with the DOX-RT-treated mice. The results of this study suggest an interaction effect on the brain in mice treated with DOX-RT. An explanation for this finding is that radiation and doxorubicin have unique mechanisms of action leading to distinct systemic effects. Doxorubicin induces DNA breaks and interrupts DNA replication via impeding the action of topoisomerases in cancer cells [34]. Doxorubicin also causes multifaceted toxic effects leading to damage in the heart, brain, liver, and kidney [34]. On the other hand, radiation therapy causes DNA breaks directly and indirectly by creating free radicals from water molecules leading to cell death [35]. Radiation therapy toxicity is seen not only at the site of irradiation in the skin but at distant sites via cytokine signaling [36]. The majority of gene changes observed were in connections to cancer gene pathways, behavior, cell stress, astrocyte activity, and neuronal transmission.

In the DOX-RT treated mice, we identified increased expression of genes associated with cognitive impairment, namely *Adar*, *Gnb1*, and *Vim*. *Adar* encodes for a protein called ADAR1, and increased expression of ADAR1 has been associated with stress-induced cognitive impairment in mice [37]. *Gnb1* expression engages in G-protein-coupled receptors by acting as a molecular switch in the signal transduction [38]. The increased expression of brain *Gnb1*, which we also observed in the DOX-only-treated mice, has been linked to anxiety and depressive behavior [39]. Doxorubicin has been shown to cause anxiety in humans and anxiety-like behavior in mice and rats [40,41,42,43]. In humans, *Gnb1* expression has been shown to be increased in neurological diseases including Alzheimer’s disease [38]. Lastly, *Vim* encodes for vimentin, which is a marker of reactive astrocytes in reactive gliosis, and its expression is increased in neurodegenerative diseases [44,45]. We previously showed reactive gliosis in multiple regions of the brain in these mice treated with DOX and DOX-RT [1].

In addition to documenting gene expression abnormalities that corroborate our previous observations of cognitive derangements in cancer treatment mice, we also identified changes in gene expression that suggest oxidative stress is a key contributor to DOX- and DOX-RT-related CRCI [1]. Specifically, increased expression of *E2f3*, *Erlec1*, *Srpr,* and *Pdgfra* in DOX-RT-treated mice implicates apoptosis, cell/endoplasmic reticulum stress, and alterations in the blood–brain barrier as pathways altered in brain tissue, although doxorubicin and hindlimb irradiation did not directly impact the brain. The *E2f3* gene encodes the e2f transcription factor 3 that is involved in DNA-damage-induced apoptosis and neurogenesis including neuronal precursor proliferation and neuronal migration [46,47]. Other researchers have previously reported doxorubicin treatment to cause increased *E2f3* mRNA and protein expression in both human and mouse cell lines [46,48]. The *Erlec1* gene encodes for endoplasmic reticulum lectin 1 and is involved in cell stress response, including endoplasmic reticulum stress, and is frequently overexpressed in human cancers [49,50,51]. Another gene associated with endoplasmic reticulum stress that was significantly increased in both the DOX and DOX-RT mice was *Srpr* [52]. In rats treated with doxorubicin, endoplasmic reticulum stress is induced in the hippocampus [43]. Increased expression of *Erlec1* and *Srpr* in the brains of the DOX-RT mice and DOX mice could indicate endoplasmic reticulum stress within the brains of the mice [51]. *Pdgfra* is an oncogene that encodes for the platelet-derived growth factor receptor alpha subunit [53]. *Pdgfra* is expressed in the hippocampus of mice, and its increased expression is linked to blood–brain barrier integrity and learning and memory [53,54]. Doxorubicin is known to cause oxidative stress within the brain that can lead to blood–brain barrier disruption [55].

We also identified the decreased expression of two genes, namely *Inpp5f* and *Rock2*, following DOX-RT treatment, which supports that abscopal effects may underlie RT-related contributions to CRCI. The *Inpp5f* gene encodes for inositol polyphosphate phosphatase F, which inhibits the PI3K/AKT signaling pathway [56]. Downregulation of the *Inpp5f* gene has been shown to be involved in neuropathic pain and cognitive impairment in rats [56]. The *Rock2* gene is highly expressed in the brain, especially within neurons. It encodes for the rho-associated coiled-coil kinase (ROCK) isoform 2 [57]. ROCK2 inhibition has been shown to reduce DNA damage repair proteins [58]. As previous studies have shown that radiation treatment can cause abscopal DNA damage, there is a possibility that the treatment could impact the repair of DNA damage in neurons affected by radiation treatment [59].

In RT-treated mice, the downregulation of *C1qa* and *C1qb* and upregulation of *Abcg2* and *Fgf2* suggests microglial and astrocyte reactivity may contribute to RT-related CRCI. *C1qa* and *C1qb* genes are two of three genes (*C1qa*, *C1qb*, and *C1qc*) that encode C1q, a protein primarily originating from microglia within the brain [60,61]. Notably, *C1qc* expression was decreased in the RT-treated mice compared to the control, but this did not reach statistical significance. Our findings of decreased *C1qa* and *C1qb* expression at 16 days post a RT are similar to those reported in study evaluating the impact of direct brain irradiation in mice. In this study, irradiation of the brain induced a transient increase in C1q expression in both astrocytes and microglia that lasted hours after treatment but dropped below control levels weeks post radiation exposure [60]. *Abcg2* encodes for ATP-binding cassette sub-family G member 2 (ABCG2), which is a transporter protein expressed in brain endothelium in the blood–brain barrier, neurons, astrocytes, microglia, and pericytes [62,63]. ABCG2 is upregulated in neurologic diseases such as Alzheimer’s disease and amyotrophic lateral sclerosis [62,64]. *Fgf2* encodes for the fibroblast growth factor 2 (FGF2) and is expressed in reactive astrocytes and neurons [65]. FGF2 has been previously shown to be upregulated after radiation treatment and is associated with radiation-induced fibrosis following cellular injury, which could contribute to cognitive dysfunction [65,66]. We showed previously that RT-treated SKH1 mice have an increased number of reactive astrocytes within multiple regions of the brain, which could explain the increased expression of *Fgf2* [1].

The DOX-only-treated mice showed significant increases in the expression of *Atf2*, *Atp5b*, and *Rad23b* and significant decreases in expression of *Sirt5* compared to control mice. *Atf2* encodes for activating transcription factor 2 (ATF2), which plays a role in normal cellular development, cellular survival, and the cellular response to stress and DNA damage [67]. Although our identification of increased *Atf2* expression aligns with similar findings from human patients with Alzheimer’s disease, Parkinson’s disease, and Huntington’s disease, it contrasts with a previous study demonstrating that mice treated with doxorubicin-based multiagent protocols had significant downregulation of brain Atf2 [8,67]. Although the precise cause for these discordant results is unknown, they may reflect differences in chemotherapy drug treatment regimes, mouse strain, and/or the timepoint at which expression in mouse brains was measured [8]. The *Atp5b* gene encodes the ATP synthase subunit beta enzyme, which is part of catalytic portion of complex V in the mitochondrial electron transport chain [68]. Our identification of increased expression of brain *Atp5b* is consistent with previous work demonstrating increased *Atp5b* and suppressed complex V activity in the hearts of doxorubicin-treated mice [68,69]. *Rad23b* is a DNA repair gene, and doxorubicin systemically damages DNA in dividing cells and can cause DNA damage to neurons [70,71,72]. This could explain why *Rad23b* was increased in the brains from DOX-treated mice. The gene *Sirt5* encodes for sirtuin 5, which resides in the mitochondria and promotes glycolysis but also has antioxidant capacity [73]. This finding corroborates a prior murine study in which doxorubicin decreased expression of *Sirt5,* which may promote doxorubicin-induced oxidative stress [74]. Doxorubicin has been shown to cause indirect oxidative stress in the nervous system, and oxidative stress can cause neuronal degeneration and cognitive impairment [72,75].

This is the first study to examine whole-brain gene expression in mice treated with concurrent doxorubicin and non-brain-directed radiation therapy or non-brain-directed radiation therapy alone. We previously demonstrated that these mice developed cognitive deficits and glial pathology following each of these treatment regimens [1]. Our data reflect gene changes associated with brain injury and cognitive dysfunction following a single post-treatment timepoint and provide novel mechanistic insights.

There are limitations to this work that can be addressed in future studies. Gene expression changes were evaluated using NanoString, which may be less sensitive to small changes in gene expression compared to RT-qPCR [76]. The effect of hormones was not evaluated in this study that used female mice only, given the predilection of women with CRCI following treatment for breast cancer. Differences in gene expression between male and female mice may shed light on unique molecular pathways for CRCI. In this study, we evaluated gene expression changes in the whole brain post treatment. There are known cognitive domains associated with CRCI that are brain-region-specific [1,19]. Additional focus is needed to study treatment-related injury to specific regions of the brain related to memory and learning. Our study investigated molecular changes after cancer treatment in a non-tumor-bearing mouse model to separate treatment-associated changes from tumor-associated changes since cancer itself causes cognitive deficits [5,9,13]. Future studies will need to evaluate the effects of cancer and anti-cancer treatments on molecular changes in the brain. Finally, all of the mice in this study underwent behavioral testing one day before the mice were euthanized by an AVMA-approved method. It is possible that both behavioral tests and euthanasia itself can alter gene expression in the brain [77,78,79,80]. Additional studies would be needed to evaluate the effect of behavioral testing and humane euthanasia methods on brain gene expression. Nonetheless, we feel that the changes in gene expression described here are important since the treatment effect was identified using similarly euthanized control mice that underwent the same behavioral testing as the treatment groups. Our study evaluated gene expression changes in mice at one timepoint translational to cancer patients years after treatment [26]. Further work will be needed to determine additional peracute and chronic changes, as our work and that of others supports a spectrum of changes that develop within hours to days and persist for weeks in rodents [1,2,21,81].

## 5. Conclusions

This study demonstrates that in adult female SKH1 mice, doxorubicin, hindlimb radiation, or concurrent doxorubicin and hindlimb radiation substantially altered gene expression patterns distinct to each treatment group. Mechanisms leading to DOX- and RT-related CRCI are unique, and the gene expression alterations identified here shed light on mitigation strategies. This study supports additional work needed to evaluate protein and gene expression changes within anatomic regions of the brain related to memory and learning following commonly used cancer treatments.

## Figures and Tables

**Table 1:** Gene differences between treatment groups. *p*-values represent results from group comparisons in a one-way ANOVA with a post hoc Tukey test. Mean = normalized mRNA counts.

Genes	Groups	Control	DOX	RT	DOX-RT
*Atp2b2*	Mean	8910	8749	9198	8298
	SEM	149.0	233.8	64.26	167.6
			DOX vs. RT	RT vs. DOX-RT	DOX-RT vs. DOX
	Tukey *p*-value		0.2512	0.0050	0.2462
	F-values	5.190			
	*p*-value	0.0082			
	DF	23			
	Number of samples	6	6	6	6
*Cd74*	Mean	123.3	126.8	104.1	166.5
	SEM	18.75	11.14	10.00	11.01
			DOX vs. RT	RT vs. DOX-RT	DOX-RT vs. DOX
	Tukey *p*-value		0.6240	0.0157	0.1783
	F-values	3.943			
	*p*-value	0.0232			
	DF	23			
	Number of samples	6	6	6	6
*Erlec1*	Mean	497.7	533.0	498.3	558.3
	SEM	14.78	8.970	8.815	8.698
			DOX vs. RT	RT vs. DOX-RT	DOX-RT vs. DOX
	Tukey *p*-value		0.1298	0.0037	0.3597
	F-values	7.634			
	*p*-value	0.0014			
	DF	23			
	Number of samples	6	6	6	6
*Gnb1*	Mean	3234	3735	3529	3986
	SEM	100.8	78.81	116.6	101.0
			DOX vs. RT	RT vs. DOX-RT	DOX-RT vs. DOX
	Tukey *p*-value		0.4826	0.0202	0.3137
	F-values	10.10			
	*p*-value	0.0003			
	DF	23			
	Number of samples	6	6	6	6
*Lyz2*	Mean	71.09	64.61	58.89	87.30
	SEM	3.388	4.631	5.420	10.26
			DOX vs. RT	RT vs. DOX-RT	DOX-RT vs. DOX
	Tukey *p*-value		0.9228	0.0264	0.0942
	F-values	3.597			
	*p*-value	0.0316			
	DF	23			
	Number of samples	6	6	6	6
*Olfml3*	Mean	81.91	77.89	65.44	84.94
	SEM	3.477	5.209	3.828	5.855
			DOX vs. RT	RT vs. DOX-RT	DOX-RT vs. DOX
	Tukey *p*-value		0.2700	0.0375	0.7152
	F-values	3.334			
	*p*-value	0.0401			
	DF	23			
	Number of samples	6	6	6	6
*Srpr*	Mean	568.2	624.2	598.0	644.8
	SEM	13.81	8.784	11.27	12.06
			DOX vs. RT	RT vs. DOX-RT	DOX-RT vs. DOX
	Tukey *p*-value		0.4047	0.0450	0.5991
	F-values	8.153			
	*p*-value	0.0010			
	DF	23			
	Number of samples	6	6	6	6
*Vim*	Mean	393.7	432.1	369.7	512.2
	SEM	23.30	21.63	9.866	46.34
			DOX vs. RT	RT vs. DOX-RT	DOX-RT vs. DOX
	Tukey *p*-value		0.4290	0.0104	0.2272
	F-values	4.781			
	*p*-value	0.0114			
	DF	23			
	Number of samples	6	6	6	6

**Table 2:** Pathway enrichment for the 17 genes differentially expressed in the brains of mice after cancer treatment.

Pathway Source	Pathway Name	*p*-Value	Adjusted *p*-Value	Odds Ratio	Combined Score	Genes Included in Pathway
*WikiPathway 2023 Human* [31,32,33]	Allograft rejection WP2328	8.724 × 10^−7^	0.0001003	71.19	993.21	*C1QB, C1QA, PDGFRA, VIM*
*KEGG 2021 Human* [31,32,33]	Human cytomegalovirus infection	9.552 × 10^−7^	0.00009552	37.43	518.83	*ATF2, PDGFRA, ROCK2, GNB1, E2F3*
*BioPlanet 2019* [31,32,33]	Melanoma	0.00002813	0.006385	62.76	657.62	*PDGFRA, E2F3, FGF2*
*KEGG 2021 Human* [31,32,33]	Melanoma	0.00002934	0.001467	61.84	645.45	*PDGFRA, E2F3, FGF2*
*Elsevier Pathway Collection* [31,32,33]	Epithelial to mesenchymal transition in cancer: overview	0.00005724	0.006158	49.00	478.69	*PDGFRA, VIM, FGF2*
*KEGG 2021 Human* [31,32,33]	Pathways in cancer	0.00006148	0.002049	15.41	149.45	*PDGFRA, ROCK2, GNB1, E2F3, FGF2*
*BioPlanet 2019* [31,32,33]	Complement activation, classical pathway	0.00009179	0.01042	177.49	1649.98	*C1QB, C1QA*
*Elsevier Pathway Collection* [31,32,33]	Proteins with altered expression in cancer metastases	0.00009322	0.006158	41.36	383.84	*PDGFRA, VIM, FGF2*
*Elsevier Pathway Collection* [31,32,33]	CR3-mediated phagocytosis in neutrophils and macrophages	0.0001032	0.006158	166.39	1527.26	*ROCK2, VIM*
*WikiPathway 2023 Human* [31,32,33]	Focal adhesion-PI3K-Akt-mTOR-signaling pathway WP3932	0.0001039	0.003050	20.33	186.43	*ATF2, PDGFRA, GNB1, FGF2*
*WikiPathway 2023 Human* [31,32,33]	Imatinib and chronic myeloid leukemia WP3640	0.0001280	0.003050	147.89	1325.55	*PDGFRA, ABCG2*
*WikiPathway 2023 Human* [31,32,33]	Spinal cord injury WP2431	0.0001314	0.003050	36.70	328.00	*C1QB, ROCK2, VIM*
*WikiPathway 2023 Human* [31,32,33]	PI3K-Akt signaling pathway WP4172	0.0001602	0.003050	18.10	158.18	*ATF2, PDGFRA, GNB1, FGF2*
*BioPlanet 2019* [31,32,33]	Angiogenesis	0.0001702	0.01288	126.74	1099.91	*PDGFRA, FGF2*
*WikiPathway 2023 Human* [31,32,33]	Complement activation WP545	0.0001702	0.003050	126.74	1099.91	*C1QB, C1QA*
*WikiPathway 2023 Human* [31,32,33]	Angiogenesis WP1539	0.0001856	0.003050	120.98	1039.39	*PDGFRA, FGF2*
*KEGG 2021 Human* [31,32,33]	PI3K-Akt signaling pathway	0.0001914	0.004784	17.26	147.77	*ATF2, PDGFRA, GNB1, FGF2*
*Elsevier Pathway Collection* [31,32,33]	Ca2+ toxicity in lens cells	0.0002357	0.01055	106.44	889.10	*PDGFRA, VIM*
*WikiPathway 2023 Human* [31,32,33]	Regulation of actin cytoskeleton WP51	0.0002603	0.003742	28.92	238.66	*PDGFRA, ROCK2, FGF2*
*BioPlanet 2019* [31,32,33]	Prion diseases	0.0003980	0.01912	80.61	631.07	*C1QB, C1QA*
*Elsevier Pathway Collection* [31,32,33]	Proteins with altered expression in cancer-associated sustaining of proliferative signaling	0.0004091	0.01454	24.68	192.55	*PDGFRA, E2F3, FGF2*
*BioPlanet 2019* [31,32,33]	Phospholipids as signaling intermediaries	0.0004212	0.01912	78.23	608.05	*PDGFRA, GNB1*
*WikiPathway 2023 Human* [31,32,33]	Microglia pathogen phagocytosis pathway WP3937	0.0005204	0.005985	69.98	529.13	*C1QB, C1QA*
*WikiPathway 2023 Human* [31,32,33]	Oxidative damage WP3941	0.0005204	0.005985	69.98	529.13	*C1QB, C1QA*
*KEGG 2021 Human* [31,32,33]	Kaposi sarcoma-associated herpesvirus infection	0.0005445	0.01089	22.32	167.77	*GNB1, E2F3, FGF2*
*BioPlanet 2019* [31,32,33]	Plasma membrane estrogen receptor signaling	0.0005468	0.02069	68.18	512.16	*ROCK2, GNB1*
*Elsevier Pathway Collection* [31,32,33]	Proteins involved in erectile dysfunction	0.0006299	0.01454	63.30	466.55	*ROCK2, FGF2*
*BioPlanet 2019* [31,32,33]	RhoA signaling pathway	0.0006589	0.02137	61.83	452.90	*ATF2, ROCK2*
*Elsevier Pathway Collection* [31,32,33]	Glioblastoma, primary	0.0007496	0.01454	57.79	415.84	*PDGFRA, FGF2*
*KEGG 2021 Human* [31,32,33]	Regulation of actin cytoskeleton	0.0007759	0.01161	19.70	141.10	*PDGFRA, ROCK2, FGF2*
*Elsevier Pathway Collection* [31,32,33]	CDH2 activation promotes cancer cell migration and survival	0.0007811	0.01454	56.56	404.65	*PDGFRA, FGF2*
*Elsevier Pathway Collection* [31,32,33]	Glioblastoma, secondary	0.0008133	0.01454	55.38	393.96	*PDGFRA, FGF2*
*BioPlanet 2019* [31,32,33]	Actin cytoskeleton regulation	0.0008612	0.02444	18.99	134.00	*PDGFRA, ROCK2, FGF2*
*KEGG 2021 Human* [31,32,33]	Coronavirus disease	0.0009291	0.01161	18.48	129.05	*C1QB, C1QA, ADAR*
*KEGG 2021 Human* [31,32,33]	Ras signaling pathway	0.0009291	0.01161	18.48	129.05	*PDGFRA, GNB1, FGF2*
*Elsevier Pathway Collection* [31,32,33]	G0/G1 cell cycle phase transition activation in cancer	0.0009481	0.01454	51.11	355.74	*PDGFRA, FGF2*
*BioPlanet 2019* [31,32,33]	ATF2 transcription factor network	0.001131	0.02851	46.61	316.24	*ATF2, PDGFRA*
*BioPlanet 2019* [31,32,33]	Glioma	0.001371	0.02851	42.16	277.93	*PDGFRA, E2F3*
*KEGG 2021 Human* [31,32,33]	Prion disease	0.001484	0.01556	15.65	101.89	*C1QB, ATF2, C1QA*
*KEGG 2021 Human* [31,32,33]	Glioma	0.001819	0.01556	36.37	229.44	*PDGFRA, E2F3*

## Data Availability

The data presented in this study are openly available in (GEO repository) at (https://www.ncbi.nlm.nih.gov/geo/query/acc.cgi?acc=GSE235567), accessed on 21 December 2023, reference number (GSE235567).
